# Efficacy and safety of the JAK1 inhibitor abrocitinib for moderate-to-severe atopic dermatitis: a systematic review and meta-analysis

**DOI:** 10.3389/fphar.2026.1735556

**Published:** 2026-07-27

**Authors:** Bobiao Ning, Baohua Li, Quan Luo, Ping Song

**Affiliations:** 1 Department of Dermatology, Xiyuan Hospital, China Academy of Chinese Medical Sciences, Beijing, China; 2 Department of Myopia Prevention and Control Center, Eye Hospital, China Academy of Chinese Medical Sciences, Beijing, China; 3 Department of Dermatology, Guangzhou Dermatology Hospital, Guangzhou, Guangdong, China

**Keywords:** abrocitinib, atopic dermatitis, janus kinase 1 inhibitor, meta-analysis, randomized controlled trial

## Abstract

**Background:**

Atopic dermatitis (AD) is a chronic inflammatory disease with substantial pruritic burden. Abrocitinib, an oral selective Janus kinase 1 (JAK1) inhibitor, targets cytokine pathways central to AD pathophysiology. This systematic review and meta-analysis evaluated its efficacy and safety in moderate-to-severe AD.

**Methods:**

Following Preferred Reporting Items for Systematic Reviews and Meta-Analyses (PRISMA) guidance, PubMed, Embase, Web of Science, and the Cochrane Library were searched to 21 August 2025 for randomized controlled trials (RCTs) comparing abrocitinib with placebo. Two reviewers independently screened studies, extracted data, and assessed risk of bias (Cochrane RoB 2.0). Pooled risk ratios (RRs) with 95% confidence intervals (CIs) were calculated; model choice was based on heterogeneity.

**Results:**

Five double-blind placebo-controlled RCTs involving 1,927 patients were included. Abrocitinib significantly improved Investigator’s Global Assessment response (RR = 2.97, 95% CI: 2.45–3.60), Eczema Area and Severity Index-75 response (RR = 2.56, 95% CI: 2.25–2.92), and ≥4-point Peak Pruritus Numerical Rating Scale improvement (RR = 2.64, 95% CI: 2.29–3.06), with greater efficacy observed for 200 mg than 100 mg. Treatment-emergent adverse events were increased (RR = 1.18, 95% CI: 1.10–1.27), mainly nausea (RR = 5.53, 95% CI: 3.41–9.34). Headache was also increased overall (RR = 1.64, 95% CI: 1.16–2.47), whereas skin allergy was reduced (RR = 0.58, 95% CI: 0.44–0.82).

**Conclusion:**

Abrocitinib provides clinically meaningful short-term improvements in disease severity and pruritus in moderate-to-severe AD. The 200 mg dose offers greater efficacy but requires careful monitoring for tolerability, particularly nausea and headache.

## 1 Introduction

Atopic dermatitis (AD), also known as atopic eczema, is a chronic, relapsing inflammatory skin disorder that significantly impairs patients’ quality of life. It is characterized by pruritus, xerosis, eczematous lesions, and a heterogeneous clinical course. Globally, AD affects up to 20% of children and approximately 3%–10% of adults, with prevalence varying across regions and age groups. The disease is associated not only with dermatological manifestations but also with systemic comorbidities, including asthma, allergic rhinitis, food allergies, anxiety, and depression, underscoring its role as a systemic immune-mediated disorder. The chronicity and psychosocial burden of moderate-to-severe AD highlight the need for effective and safe long-term treatment strategies. Pathophysiologically, AD is primarily driven by dysregulation of the type 2 immune response (Edwards et al., 2024; Chovatiya and Paller, 2021; Napolitano et al., 2022). Cytokines such as interleukin (IL)-4, IL-13, IL-22, and IL-31 play central roles in disease initiation and persistence through their effects on skin barrier function, immune cell activation, and pruritus signaling. Janus kinase (JAK)–signal transducer and activator of transcription (STAT) pathways are critical mediators of these cytokine signals. This mechanistic understanding has paved the way for targeted therapies, particularly small-molecule JAK inhibitors, which offer oral alternatives to biologics by modulating multiple cytokine pathways simultaneously (Reich et al., 2023; Iznardo et al., 2023; Nakashima et al., 2022).

Abrocitinib, an oral selective JAK1 inhibitor, has emerged as a promising therapeutic option for patients with moderate-to-severe AD who are inadequately controlled with conventional systemic immunosuppressants or topical therapies. By selectively inhibiting JAK1, abrocitinib disrupts signaling pathways of key type 2 cytokines, thereby reducing inflammation and pruritus. Clinical development programs have included multiple phase II and phase III randomized controlled trials (RCTs), such as JADE MONO, JADE TEEN, JADE COMPARE, and JADE EXTEND, which collectively evaluated abrocitinib across diverse patient populations. These trials reported significant improvements in validated endpoints, including the Investigator’s Global Assessment (IGA), Eczema Area and Severity Index (EASI), and Peak Pruritus Numerical Rating Scale (PP-NRS) (Kim et al., 2024; Huang et al., 2022). Additionally, abrocitinib demonstrated a rapid onset of action, particularly in pruritus reduction, which is a primary driver of disease burden. Despite its therapeutic benefits, concerns regarding safety and tolerability remain. Reported adverse events (AEs) include nausea, headache, acne, and infections, while laboratory abnormalities such as thrombocytopenia and lipid elevations have also been observed. Regulatory agencies, including the European Medicines Agency (EMA) and the U.S. Food and Drug Administration (FDA), have recommended careful monitoring of abrocitinib’s safety profile, particularly in relation to thromboembolic events and serious infections, risks shared with other JAK inhibitors. Given the heterogeneity of trial designs, populations, and dosing regimens, a comprehensive synthesis of available evidence is warranted to clarify both the efficacy and safety profiles of abrocitinib (Li et al., 2025; Li et al., 2023; Pareek et al., 2024).

Therefore, this systematic review and meta-analysis aims to comprehensively evaluate the efficacy and safety of abrocitinib in the treatment of moderate-to-severe AD. By integrating data from available randomized controlled trials, we seek to provide high-level evidence regarding its therapeutic benefits, onset of action, tolerability, and potential risks, thereby supporting clinical decision-making and informing future guidelines for AD management.

## 2 Methods

### 2.1 Search strategy

This systematic review and meta-analysis was conducted in accordance with the Preferred Reporting Items for Systematic Reviews and Meta-Analyses (PRISMA) guidelines (Page et al., 2021). A comprehensive literature search was performed in PubMed, Embase, Web of Science, and the Cochrane Library from their inception to 21 August 2025, to identify randomized controlled trials (RCTs) evaluating the efficacy and safety of abrocitinib in patients with moderate-to-severe atopic dermatitis. No restrictions were applied regarding language or publication status. Non-English articles were considered if an English abstract was available. To ensure completeness, we also manually searched the reference lists of all relevant articles, systematic reviews, and meta-analyses for additional eligible studies. Grey literature, including conference abstracts and trial registry records (e.g., ClinicalTrials.gov, WHO International Clinical Trials Registry Platform), was screened when relevant. The search strategy combined Medical Subject Headings (MeSH) terms and free-text keywords. The primary terms included “abrocitinib”, “JAK1 inhibitor”, “Janus kinase one inhibitor”, and terms related to the condition of interest such as “atopic dermatitis”, “eczema”, “moderate-to-severe atopic dermatitis”, and “chronic eczema”. These terms were used individually and in various combinations with Boolean operators (“AND”, “OR”) to maximize sensitivity. Search filters were restricted to randomized controlled trials. The search strategy is presented in Supplementary Table 1.

### 2.2 Inclusion criteria and exclusion criteria

Studies were considered eligible if they met the following criteria:

1. Study design: Randomized controlled trials (RCTs) that evaluated the efficacy and/or safety of abrocitinib in the treatment of atopic dermatitis.
2. Population: Patients diagnosed with moderate-to-severe atopic dermatitis, irrespective of age, sex, or geographic region. Diagnostic criteria were required to be clearly defined according to recognized clinical or dermatological guidelines.
3. Intervention: Abrocitinib administered as monotherapy or in combination with other therapies.
4. Comparator: Placebo or other active treatments, including topical or systemic agents.
5. Outcomes: Studies that reported at least one efficacy outcome (e.g., Eczema Area and Severity Index [EASI], Investigator’s Global Assessment [IGA], Peak Pruritus Numerical Rating Scale [PP-NRS]) or safety outcome (e.g., incidence of treatment-emergent adverse events, serious adverse events, or laboratory abnormalities).

Studies were excluded if they met any of the following criteria:

1. Non-randomized studies, observational studies, case reports, case series, reviews, editorials, or letters without original data.
2. Trials that did not include abrocitinib as an intervention.
3. Studies with insufficient data for quantitative synthesis, even after attempts to contact authors for clarification.
4. Duplicate publications or overlapping data; in such cases, the most complete or recent dataset was included.

### 2.3 Literature screening and data extraction

Two investigators independently screened and extracted data from the included studies according to the predefined eligibility criteria. Titles and abstracts were initially reviewed to exclude irrelevant publications, and the full texts of potentially eligible studies were subsequently assessed to determine final inclusion. Any discrepancies between the two reviewers were resolved through discussion, and a third investigator was consulted when consensus could not be reached. For each eligible study, data were extracted using a standardized data collection form. The following information was recorded: first author, year of publication, study design, intervention and comparator groups, details of the therapeutic regimen (including dose and duration), sample size, demographic and baseline characteristics of participants, follow-up period, and reported clinical outcomes. Efficacy outcomes included commonly used indices such as the EASI, IGA, and PP-NRS. Safety outcomes included treatment-emergent adverse events (TEAEs), serious adverse events (SAEs), laboratory abnormalities, and discontinuations due to adverse events. For safety outcomes, adverse events were extracted according to the terminology used in the original trials. “Skin allergy” was defined as cutaneous allergic or hypersensitivity-related adverse events reported by the included studies, including rash, erythema, urticaria, allergic dermatitis, eczema-like eruption, or pruritic erythematous lesions when reported under comparable categories.

### 2.4 Quality assessment

The methodological quality and risk of bias of the included randomized controlled trials were independently assessed by two reviewers using the Cochrane Risk of Bias Tool (RoB 2.0) (Sterne et al., 2019). The following domains were evaluated: (1) bias arising from the randomization process, (2) bias due to deviations from intended interventions, (3) bias due to missing outcome data, (4) bias in measurement of outcomes, and (5) bias in selection of the reported results. Each domain was judged as “low risk of bias,” “some concerns,” or “high risk of bias.” Disagreements between reviewers were resolved through discussion, and a third investigator was consulted when consensus could not be achieved. A risk-of-bias summary was generated to provide a visual representation of the overall quality of evidence across studies. The results of the quality assessment were used to inform the interpretation of findings and to conduct sensitivity analyses when necessary.

### 2.5 Statistical analyses

All statistical analyses were performed in accordance with the recommendations of the Cochrane Handbook for Systematic Reviews of Interventions. Data were synthesized using Stata (version 18.0; StataCorp, College Station, TX, USA). For dichotomous outcomes, pooled effect estimates were calculated as risk ratios (RRs) with corresponding 95% confidence intervals (CIs). A p-value <0.05 was considered statistically significant. Statistical heterogeneity among studies was assessed using the Cochran’s Q test (with p < 0.10 indicating significant heterogeneity) and quantified with the I^2^ statistic. An I^2^ value of 25%–50% was considered low heterogeneity, 50%–75% moderate heterogeneity, and >75% high heterogeneity. A fixed-effect model was applied when heterogeneity was negligible; otherwise, a random-effects model (DerSimonian and Laird method) was used to provide more conservative estimates. Subgroup analyses were conducted based on abrocitinib dosages, to explore potential sources of heterogeneity. Sensitivity analyses were performed by sequentially excluding individual studies to assess the robustness of the pooled results. Potential publication bias was evaluated by visual inspection of funnel plots when at least ten studies were included in the analysis. In addition, Begg’s rank correlation test and Egger’s regression test were applied to formally assess small-study effects.

## 3 Results

### 3.1 Search results and study selection

**FIGURE 1:**
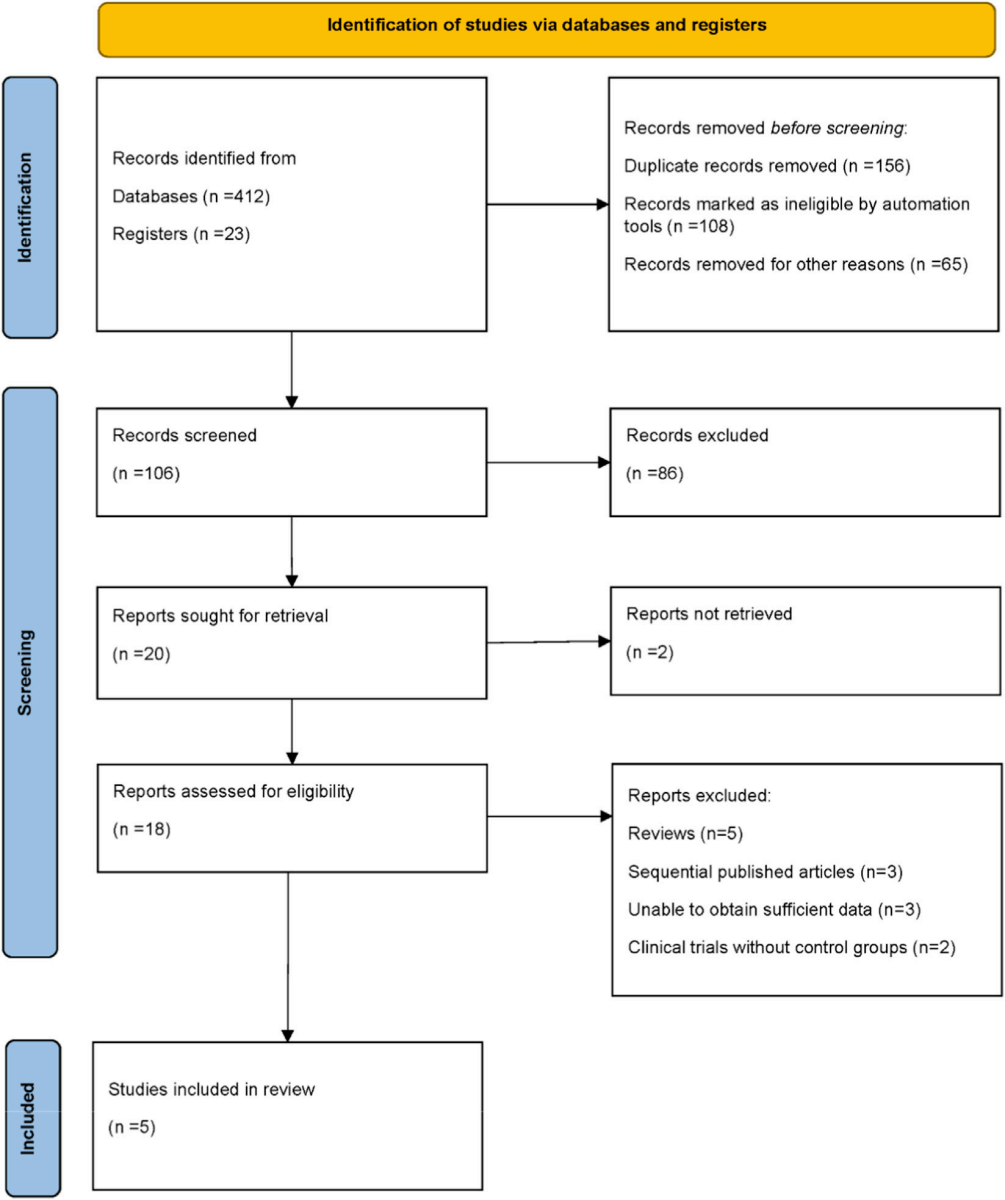
PRISMA flow diagram showing the selection of five randomized controlled trials from 435 records.

A total of 435 records were identified through the initial database and registry searches, including 412 from electronic databases and 23 from trial registers. After removing 156 duplicate records, 108 records marked as ineligible by automated tools, and 65 records excluded for other reasons, 106 records remained for screening. Following title and abstract screening, 86 records were excluded, leaving 20 reports for retrieval. Among these, two reports could not be retrieved. The remaining 18 reports were assessed for eligibility through full-text review. Of these, 10 reports were excluded: five were reviews, three were sequentially published articles, three lacked sufficient data for analysis, and two were clinical trials without control groups. Finally, five randomized controlled trials met the inclusion criteria and were included in the qualitative and quantitative synthesis of this systematic review and meta-analysis (Bieber et al., 2021; Eichenfield et al., 2021; Gooderham et al., 2019; Silverberg et al., 2020; Simpson et al., 2020). The detailed selection process is illustrated in Figure 1.

### 3.2 Study characteristics

Five randomized controlled trials were included in this meta-analysis, encompassing a total of 1,927 patients with moderate-to-severe atopic dermatitis. All studies were double-blind, placebo-controlled trials that investigated the efficacy and safety of abrocitinib. The sample sizes of individual studies ranged from 267 to 595 participants, and the enrolled populations included both adolescents (12–17 years) and adults (≥18 years). Eligibility criteria were consistent across studies, requiring patients to present with an IGA score of at least three and an EASI score of ≥16 in most trials, with one study applying a slightly lower threshold of EASI ≥12. Treatment duration varied from 12 to 16 weeks. Intervention groups received oral abrocitinib at doses of either 100 mg or 200 mg once daily, while control groups received placebo (Table 1).

**TABLE 1:** Characteristics of included randomized controlled trials on abrocitinib in moderate-to-severe atopic dermatitis.

First author	Year	Study design	Sample size (n)	Age range (years)	IGA criteria	EASI criteria	Duration (weeks)	Intervention dose (mg, qd)	Comparator
Eichenfield	2021	RCT	287	12–17	≥3	≥16	12	100/200	Placebo
Bieber	2021	RCT	595	≥18	≥3	≥16	16	100/200	Placebo
Silverberg	2020	RCT	391	≥12	≥3	≥16	12	100/200	Placebo
Simpson	2020	RCT	387	≥12	≥3	≥16	12	100/200	Placebo
Gooderham	2019	RCT	267	≥18	≥3	≥12	12	100/200	Placebo

IGA, Investigator’s Global Assessment.

EASI, Eczema Area and Severity Index.

RCT, Randomized Controlled Trial.

qd, once daily.

### 3.3 Results of quality assessment

**FIGURE 2:**
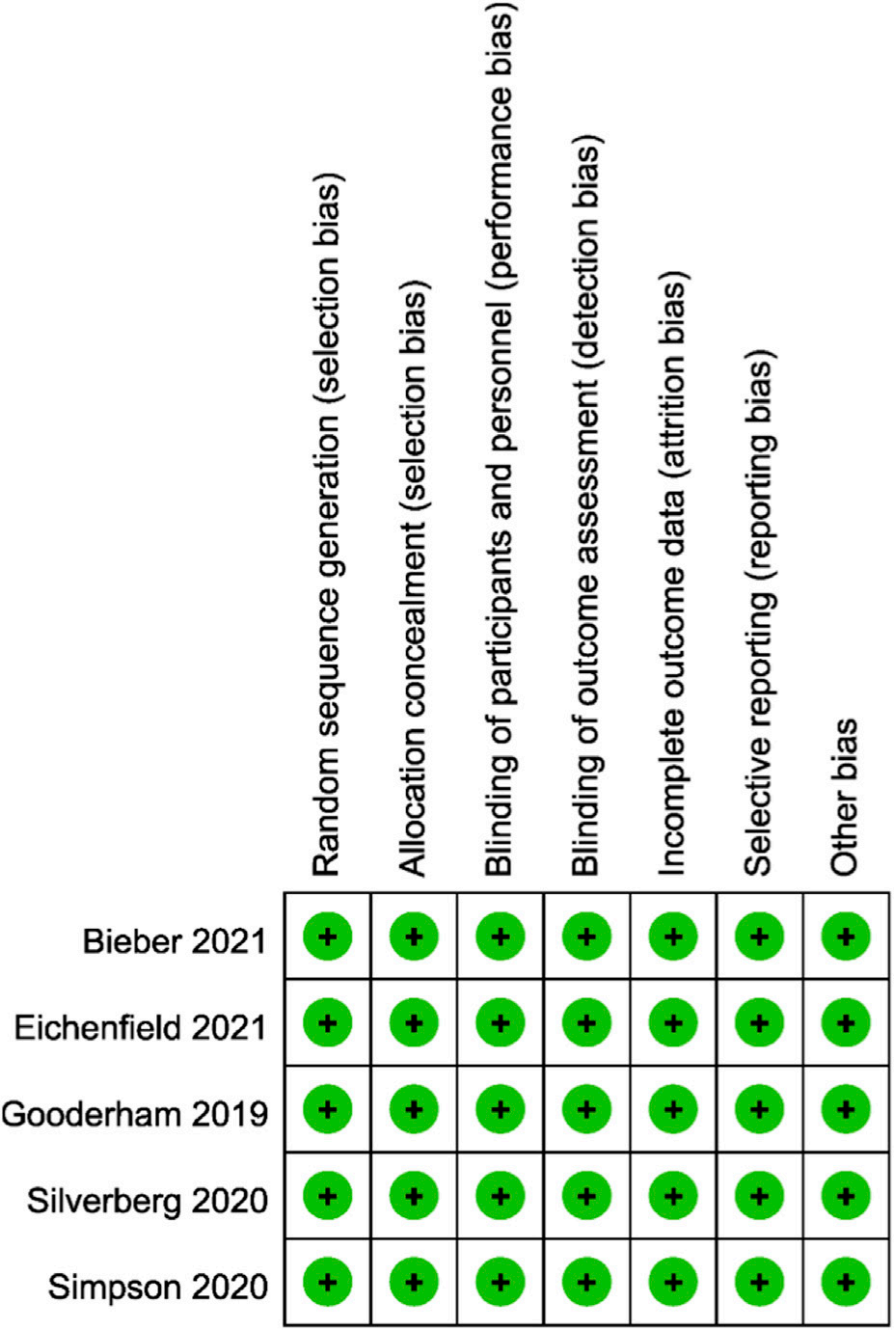
Risk-of-bias assessment of included trials using the Cochrane RoB 2.0 tool, indicating overall low risk.

The methodological quality of the included randomized controlled trials was generally high. All studies adequately reported methods of random sequence generation and allocation concealment, indicating a low risk of selection bias. Blinding of participants, study personnel, and outcome assessors was consistently maintained, minimizing the risk of performance and detection bias. Incomplete outcome data were appropriately addressed, and all studies provided clear reporting of prespecified outcomes, suggesting a low risk of attrition and reporting bias. No additional sources of bias were identified. Overall, the risk of bias across the included trials was judged to be low, supporting the reliability and robustness of the pooled findings in this meta-analysis (Figure 2).

### 3.4 Improvement in investigator’s global assessment

**FIGURE 3:**
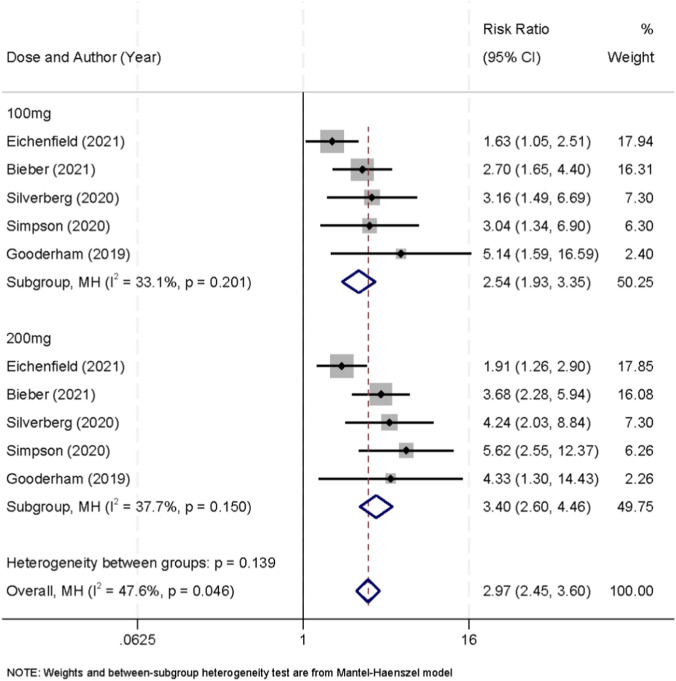
Forest plot showing abrocitinib significantly improved IGA response (≥2-point reduction) versus placebo.

All five randomized controlled trials reported the proportion of patients who achieved a reduction of at least two points from baseline in the IGA score. No significant heterogeneity was observed across studies (I^2^ < 50%, p > 0.10); therefore, a fixed-effects model was applied for pooled analysis. The meta-analysis demonstrated that abrocitinib significantly increased the proportion of patients achieving a ≥2-point reduction in IGA score compared with placebo (RR = 2.97, 95% CI: 2.45–3.60, p < 0.001). This finding indicates that patients receiving abrocitinib were nearly three times more likely to experience clinically meaningful improvement in disease severity than those receiving placebo. Subgroup analyses based on dosage revealed consistent benefits at both 100 mg and 200 mg once daily. Specifically, the 100 mg group showed a significantly higher response rate compared with placebo (RR = 2.54, 95% CI: 1.93–3.35, p < 0.001), while the 200 mg group demonstrated an even greater treatment effect (RR = 3.40, 95% CI: 2.60–4.46, p < 0.001). These results suggest a dose-response relationship, with the higher abrocitinib dose associated with a greater likelihood of achieving IGA improvement (Figure 3).

### 3.5 Improvement in peak pruritus numerical rating scale

**FIGURE 4:**
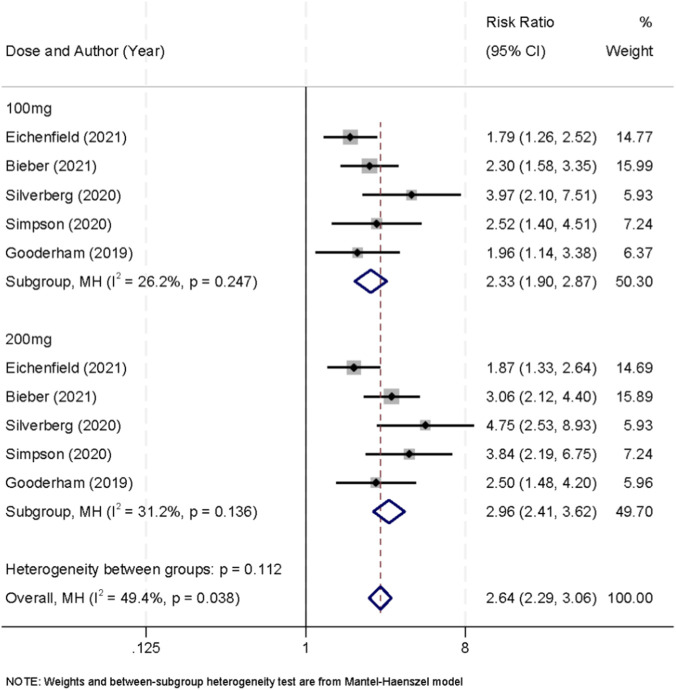
Forest plot showing abrocitinib significantly increased PP-NRS improvement (≥4-point reduction) compared with placebo.

All five randomized controlled trials assessed the proportion of patients who achieved a clinically meaningful reduction of at least four points from baseline in the PP-NRS. This outcome is recognized as a validated measure of itch relief and is considered a clinically relevant endpoint in atopic dermatitis research. No significant heterogeneity was observed among the included studies (I^2^ < 50%, p > 0.10); therefore, a fixed-effects model was applied. The pooled analysis demonstrated that patients receiving abrocitinib were significantly more likely to achieve a ≥4-point improvement in PP-NRS compared with placebo (RR = 2.64, 95% CI: 2.29–3.06, p < 0.001). This finding indicates that abrocitinib substantially alleviated pruritus, which is one of the most burdensome symptoms of atopic dermatitis and strongly associated with impaired quality of life. Subgroup analyses further revealed consistent benefits across both dosing regimens. At a daily dose of 100 mg, abrocitinib was associated with a markedly higher likelihood of achieving a clinically meaningful PP-NRS improvement relative to placebo (RR = 2.33, 95% CI: 1.90–2.87, p < 0.001). The 200 mg dose demonstrated an even stronger effect, with nearly a threefold increase in response rates compared with placebo (RR = 2.96, 95% CI: 2.41–3.62, p < 0.001). These results highlight a clear dose-response relationship, with the higher abrocitinib dose producing superior antipruritic efficacy (Figure 4).

### 3.6 Achievement of EASI-75 response

**FIGURE 5:**
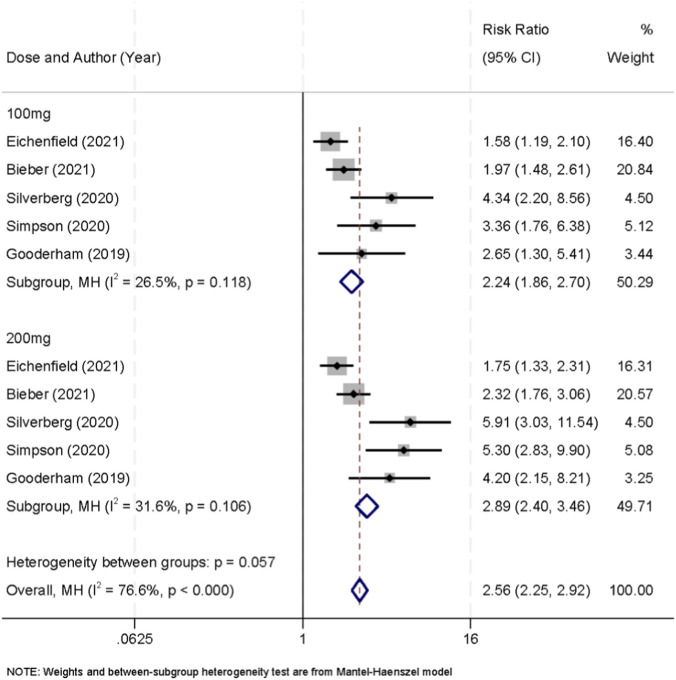
Forest plot showing abrocitinib significantly improved EASI-75 response compared with placebo.

All five included randomized controlled trials reported the proportion of patients achieving at least a 75% improvement from baseline in the EASI-75, which is widely regarded as a clinically meaningful endpoint in atopic dermatitis trials. No significant heterogeneity was observed across the studies (I^2^ < 50%, p > 0.10), and therefore a fixed-effects model was applied for pooled analysis. The meta-analysis demonstrated that abrocitinib treatment was associated with a significantly higher likelihood of achieving EASI-75 compared with placebo (RR = 2.56, 95% CI: 2.25–2.92, p < 0.001). This indicates that patients receiving abrocitinib were more than twice as likely to experience substantial improvement in skin lesions relative to placebo. Subgroup analyses stratified by dose revealed consistent benefits across both 100 mg and 200 mg once-daily regimens. Specifically, the 100 mg dose significantly increased the probability of achieving EASI-75 compared with placebo (RR = 2.24, 95% CI: 1.86–2.70, p < 0.001), while the 200 mg dose demonstrated an even greater therapeutic effect (RR = 2.89, 95% CI: 2.40–3.46, p < 0.001). These findings suggest a dose-dependent relationship, with the higher dose conferring greater efficacy in achieving marked clinical improvement (Figure 5).

### 3.7 Overall incidence of adverse events

**FIGURE 6:**
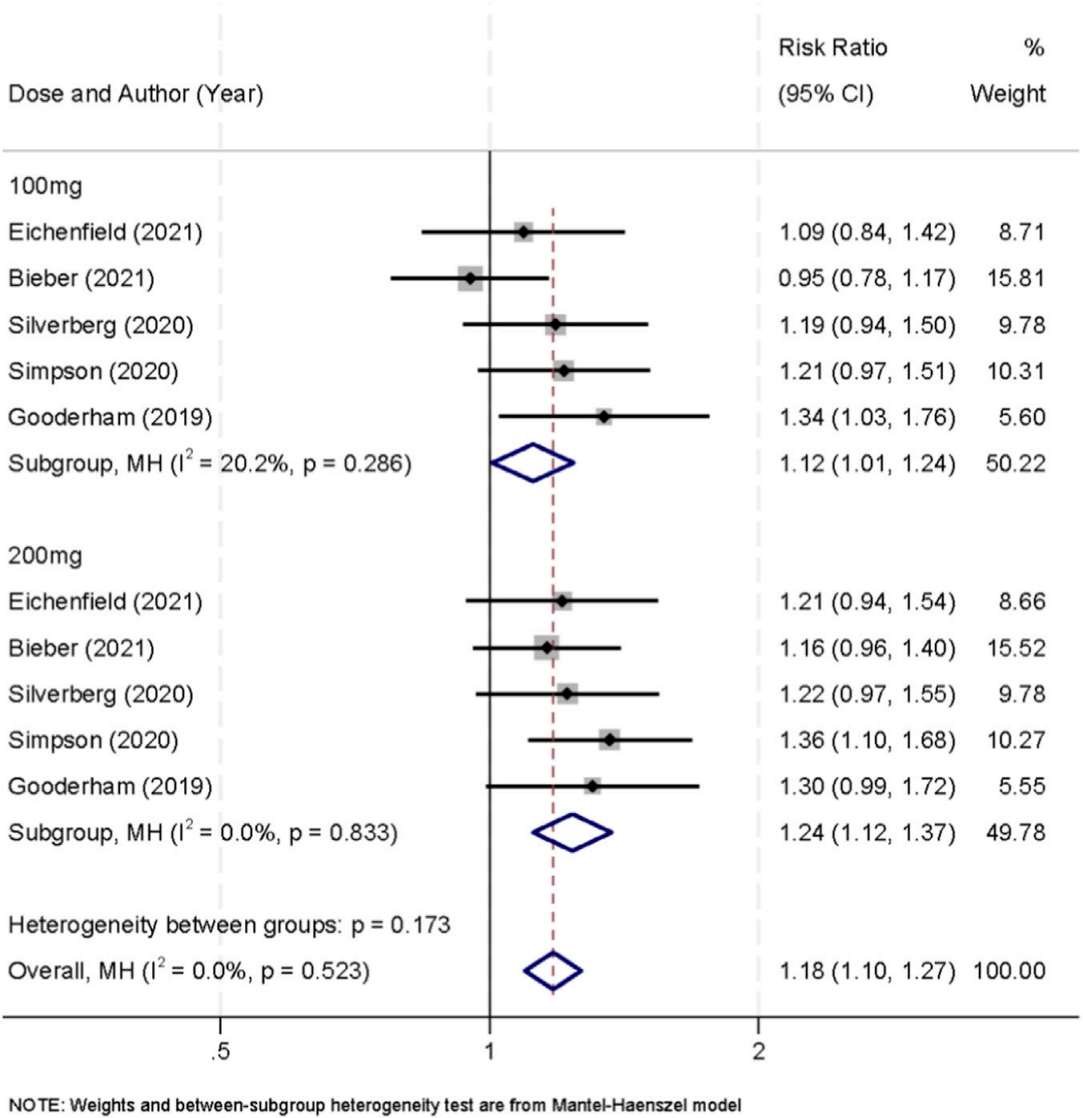
Forest plot showing abrocitinib was associated with a higher overall incidence of treatment-emergent adverse events.

All five randomized controlled trials reported the overall incidence of TEAEs in patients receiving abrocitinib and placebo. No significant heterogeneity was observed among the included studies (I^2^ < 50%, p > 0.10); therefore, a fixed-effects model was used for pooled analysis. The meta-analysis revealed that the overall risk of adverse events was significantly higher in the abrocitinib groups compared with placebo (RR = 1.18, 95% CI: 1.10–1.27, p < 0.001). This suggests that while abrocitinib provides substantial clinical benefits, it is also associated with an increased likelihood of experiencing treatment-related adverse events. Subgroup analyses stratified by dosage demonstrated that both 100 mg and 200 mg daily regimens were associated with a statistically significant increase in adverse event incidence relative to placebo. Specifically, the 100 mg group showed a modest but significant elevation in TEAEs (RR = 1.12, 95% CI: 1.01–1.24, p < 0.001), while the 200 mg group exhibited a more pronounced risk (RR = 1.24, 95% CI: 1.12–1.37, p < 0.001) (Figure 6).

### 3.8 Adverse events

All five randomized controlled trials reported treatment-emergent adverse events associated with abrocitinib compared with placebo. Abrocitinib was associated with a significantly higher risk of nausea in the overall comparison (RR = 5.53, 95% CI: 3.41–9.34, p < 0.0001), with significant increases observed for both the 100 mg dose (RR = 3.30, 95% CI: 1.53–6.75, p = 0.002) and the 200 mg dose (RR = 8.23, 95% CI: 3.94–16.04, p < 0.0001). Headache was also significantly increased in the overall comparison between abrocitinib and placebo (RR = 1.64, 95% CI: 1.16–2.47, p = 0.009). In contrast, abrocitinib was associated with a significantly lower risk of skin allergy in the overall comparison (RR = 0.58, 95% CI: 0.44–0.82, p = 0.001), particularly in the 200 mg group (RR = 0.40, 95% CI: 0.25–0.68, p < 0.001) (Table 2).

**TABLE 2:** Summary of adverse events in patients receiving abrocitinib versus placebo.

Treatment comparison	Adverse event	No. of studies	No. of cases	I^2^ (%)	Model	RR (95% CI)	P value
Abrocitinib (100 mg, qd) vs. placebo	Nausea	5	1,127	0	Fixed	3.30 (1.53–6.75)	0.002
Abrocitinib (100 mg, qd) vs. placebo	Nasopharyngitis	4	1,020	0	Fixed	1.38 (0.93–2.07)	0.120
Abrocitinib (100 mg, qd) vs. placebo	Headache	5	1,127	0	Fixed	1.44 (0.82–2.42)	0.237
Abrocitinib (100 mg, qd) vs. placebo	Upper respiratory tract infection	5	1,127	0	Fixed	1.10 (0.69–1.77)	0.661
Abrocitinib (100 mg, qd) vs. placebo	Skin allergy	4	1,127	0	Fixed	0.74 (0.49–1.08)	0.131
Abrocitinib (200 mg, qd) vs. placebo	Nausea	5	1,103	0	Fixed	8.23 (3.94–16.04)	<0.0001
Abrocitinib (200 mg, qd) vs. placebo	Nasopharyngitis	4	1,018	0	Fixed	1.15 (0.76–1.77)	0.489
Abrocitinib (200 mg, qd) vs. placebo	Headache	5	1,103	0	Fixed	1.01 (0.63–1.59)	0.989
Abrocitinib (200 mg, qd) vs. placebo	Upper respiratory tract infection	5	1,103	0	Fixed	1.00 (0.61–1.57)	0.976
Abrocitinib (200 mg, qd) vs. placebo	Skin allergy	4	753	0	Fixed	0.40 (0.25–0.68)	0.000
Abrocitinib vs. placebo	Nausea	5	2,230	0	Fixed	5.53 (3.41–9.34)	<0.0001
Abrocitinib vs. placebo	Nasopharyngitis	4	2038	0	Fixed	1.27 (0.94–1.68)	0.109
Abrocitinib vs. placebo	Headache	5	2,230	0	Fixed	1.64 (1.16–2.47)	0.009
Abrocitinib vs. placebo	Upper respiratory tract infection	5	2,230	0	Fixed	1.05 (0.62–1.54)	0.756
Abrocitinib vs. placebo	Skin allergy	4	1880	0	Fixed	0.58 (0.44–0.82)	0.001

### 3.9 Publication bias

Publication bias was assessed using Egger’s linear regression test for the primary efficacy and safety outcomes. The results indicated no evidence of significant publication bias, as all p-values from Egger’s test were greater than 0.05.

## 4 Discussion

This meta-analysis synthesized evidence from five RCTs to evaluate the efficacy and safety of the JAK1 inhibitor abrocitinib for moderate-to-severe AD. Across trials, abrocitinib conferred consistent improvements in clinically validated endpoints. Patients receiving abrocitinib were nearly three times as likely as those receiving placebo to achieve an IGA response (≥2-point reduction), and were more than twice as likely to reach EASI-75. Antipruritic efficacy was robust, with a ≥4-point improvement in PP-NRS observed significantly more often in the abrocitinib arms. Each endpoint demonstrated a clear dose–response gradient favoring 200 mg over 100 mg once daily. These effects align mechanistically with selective JAK1 inhibition, which modulates signaling by type-2 cytokines implicated in barrier dysfunction and itch neurobiology in AD. The trade-off for greater efficacy was a modest elevation in TEAEs, most notably dose-related nausea. Headache was also increased in the overall comparison, although dose-specific analyses did not individually reach statistical significance. In contrast, the risks of nasopharyngitis and upper respiratory tract infection did not differ significantly from placebo. Altogether, the evidence supports abrocitinib as an effective oral systemic option for patients with moderate-to-severe AD, with tolerability considerations that are dose dependent.

The magnitude and consistency of effects across IGA, EASI-75, and PP-NRS indicate that abrocitinib improves both objective disease activity and patient-reported symptoms. The near-threefold increase in IGA responders reflects clinically relevant disease control and parallels gains in lesion clearance captured by EASI-75. Importantly, the antipruritic effect, central to AD burden, was large and dose-responsive, supporting a role for abrocitinib in rapidly mitigating itch. The observed gradient between 100 mg and 200 mg is clinically informative, suggesting that the higher dose may provide greater benefit for patients with high disease activity or refractory pruritus, provided that safety is carefully monitored. The absence of between-study heterogeneity for these endpoints supports the statistical consistency of the estimates within this closely related trial program, although this should be interpreted in the context of highly homogeneous trial designs and populations. The TEAE risk ratio was modestly elevated versus placebo, with a larger effect at 200 mg than 100 mg, indicating dose dependence. Nausea drove much of this signal and was the only adverse event with a consistently higher risk across dose strata. Headache also showed a significant increase in the overall comparison, whereas dose-specific estimates did not reach statistical significance. For nasopharyngitis and upper respiratory tract infection, no statistically significant increase was observed in this analysis; however, the relatively wide confidence intervals for some infection outcomes indicate that a potential difference in infection risk cannot be completely ruled out. Skin allergy events were lower in abrocitinib-treated patients, particularly in the 200 mg group. Clinically, these events are generally managed according to severity, including emollients, topical corticosteroids, oral antihistamines, close monitoring, and treatment interruption or discontinuation when severe or systemic hypersensitivity is suspected. These findings suggest that the short-term safety profile is mainly characterized by predictable and largely non-serious tolerability issues, especially nausea and headache, while longer-term real-world data are needed to further evaluate infection risk. From a practical perspective, anticipatory counseling about nausea, headache, and early dose-related tolerability issues may improve adherence and outcomes. Serious adverse events of special interest, including major adverse cardiovascular events (MACE) and venous thromboembolism (VTE), should also be considered when interpreting abrocitinib safety. Although these events were uncommon in the abrocitinib clinical development program, the 12–16-week placebo-controlled trials were not sufficient to estimate their risks precisely. Therefore, baseline cardiovascular and thrombotic risk assessment, long-term extension data, real-world evidence, and continued pharmacovigilance remain necessary, particularly in patients with cardiovascular disease, prior VTE, or other thrombotic risk factors.

Recent extension and synthesis studies provide convergent support for the efficacy and longer-term tolerability observed here. In the phase three long-term extension program (JADE EXTEND), efficacy and patient-reported outcomes with abrocitinib were maintained up to 48–96 weeks, with no new safety signals, corroborating the durability and safety consistency of JAK1 inhibition beyond the 12–16-week RCT window assessed in our meta-analysis (Reich et al., 2023; Silverberg et al., 2023). Updated integrated safety analyses through nearly 4 years of exposure similarly reported a stable profile without emergent risks, while continuing to identify nausea as a frequent, usually manageable event; these data align with our dose-dependent TEAE findings (Simpson et al., 2024). Narrative and systematic reviews published in 2024–2025, spanning clinical trials and observational cohorts, have reiterated abrocitinib’s rapid antipruritic effect and broad efficacy across dermatitis severity strata, consistent with our pooled PP-NRS and EASI-75 results (Dogra et al., 2024; Nezamololama et al., 2020; Bieber et al., 2022). Comparative evidence also informs positioning among targeted systemic agents. A recent network meta-analysis of JAK inhibitors concluded that upadacitinib 30 mg often ranks highest on efficacy endpoints, although abrocitinib performs robustly across IGA, EASI, and itch measures; our pooled effect sizes for abrocitinib are concordant with that ranking, and the within-drug dose gradient we observed parallels class-level dose–response patterns (Kim et al., 2025). Real-world registry and cohort data, including multicenter European studies, have documented effectiveness and a safety profile dominated by non-serious events with nausea frequently reported, observations that mirror our trial-based synthesis and support external validity (Kamphuis et al., 2024). Finally, subpopulation analyses, adolescents in particular, have shown sustained benefits with abrocitinib exposure in extension cohorts, aligning with our mixed adolescent–adult RCT dataset and suggesting applicability across the upper pediatric and adult age spectrum (Ferreira et al., 2020).

Our findings should be interpreted in the context of prior evidence, particularly the meta-analysis by Li et al. (Li et al., 2023), which evaluated the efficacy and safety of abrocitinib in adolescents and adults with moderate-to-severe atopic dermatitis. The present study adds incremental value by providing an updated and more focused synthesis of double-blind placebo-controlled randomized controlled trials, integrating clinically interpretable efficacy endpoints, including IGA response, EASI-75 response, and ≥4-point PP-NRS improvement. We also examined dose-stratified efficacy and safety for the approved 100 mg and 200 mg regimens, allowing a clearer assessment of the efficacy–tolerability balance. In addition, our analysis updated the safety interpretation by highlighting the overall increase in headache, the dose-dependent increase in nausea, and the residual uncertainty regarding infection-related outcomes due to relatively wide confidence intervals. These refinements provide a more clinically oriented synthesis that supports individualized dose selection and practical considerations for abrocitinib use. Taken together, contemporary evidence corroborates the principal findings of this meta-analysis: abrocitinib yields clinically meaningful, dose-related improvements in skin clearance and itch, with a tolerability profile in which nausea predominates and serious safety events appear infrequent under trial conditions (Reich et al., 2023; Armario-Hita et al., 2023). Nevertheless, although no statistically significant increase in nasopharyngitis or upper respiratory tract infection was observed in our analysis, the relatively wide confidence intervals for some infection-related outcomes indicate remaining uncertainty. Therefore, longer-term real-world data and continued pharmacovigilance are needed to further evaluate infection risk in broader clinical populations. Abrocitinib offers an oral, once-daily alternative to injectable biologics for patients with moderate-to-severe AD who require systemic therapy. The 200 mg dose provides a greater probability of Investigator’s Global Assessment response, Eczema Area and Severity Index-75 attainment, and itch reduction, but it also increases the likelihood of nausea. Therefore, dose selection should incorporate disease activity, patient priorities, infection-related risk factors, and tolerability. Shared decision-making should include counseling on early-onset nausea and planned mitigation strategies, including timing of administration, supportive care, and temporary dose adjustment. In settings where rapid antipruritic benefit is prioritized, abrocitinib represents a rational systemic option, provided that safety monitoring is individualized (Dogra et al., 2024; Butala et al., 2023).

This meta-analysis has several strengths. First, it included only randomized, placebo-controlled trials, which reduces confounding and enhances internal validity. Second, endpoints were prespecified, clinically validated, and complementary, spanning physician-assessed severity (IGA), composite lesion burden (EASI-75), and PP-NRS. Third, the absence of statistical heterogeneity across primary efficacy outcomes supports the stability of pooled effects. Fourth, prespecified dose-stratified analyses allowed quantification of a clinically relevant dose–response relationship. Several limitations should be considered. First, all included randomized controlled trials were derived from the abrocitinib clinical development program, including the JADE trials. These studies shared highly similar designs, eligibility criteria, placebo-controlled comparisons, dosing regimens, outcome definitions, and follow-up durations. Therefore, the consistently low statistical heterogeneity, including I^2^ values of 0% for several outcomes, should not be overinterpreted as evidence of broad consistency across fully independent studies. Rather, it may primarily reflect the homogeneous trial designs and patient populations. In this context, the present analysis should be regarded as a trial-level pooled synthesis of closely related randomized controlled trials, and the generalizability of the pooled estimates to broader clinical settings remains uncertain. Second, trial populations were enriched for patients eligible for systemic therapy and may under-represent multimorbid patients, older individuals, patients with complex comorbidities, and more diverse real-world populations, thereby limiting external validity. Third, safety outcome definitions and ascertainment were not fully standardized across trials, and the short follow-up limited precise evaluation of uncommon or delayed adverse events, including major adverse cardiovascular events, venous thromboembolism, opportunistic infections, malignancy, and clinically relevant laboratory abnormalities. Although nasopharyngitis and upper respiratory tract infection were not significantly increased, the relatively wide confidence intervals for some infection-related outcomes indicate residual uncertainty. Therefore, longer-term extension studies, real-world cohort data, and continued pharmacovigilance are needed as cumulative exposure increases in broader clinical practice.

## 5 Conclusion

Abrocitinib significantly improved disease severity and pruritus in moderate-to-severe atopic dermatitis, with greater efficacy observed at 200 mg than at 100 mg. Overall TEAEs were modestly increased, mainly due to nausea, and headache was also increased in the overall comparison, whereas common infections were comparable to placebo. Abrocitinib represents an effective oral option, but dose selection should balance efficacy and tolerability with early monitoring.

## Data Availability

The original contributions presented in the study are included in the article/Supplementary Material, further inquiries can be directed to the corresponding author.
