# Supplementary material for: Efficacy and safety of the JAK1 inhibitor abrocitinib for moderate-to-severe atopic dermatitis: a systematic review and meta-analysis
Source: Front Pharmacol. 2026 Jul 27;17:1735556. doi: 10.3389/fphar.2026.1735556 (PMC13454108; doi:10.3389/fphar.2026.1735556)
Supplement: Supplementary file 1 [file Table1.docx]

Supplementary Table 1. Detailed Search Strategies for Each Database.

| Database | Search Strategy |
| --- | --- |
| PubMed | (((("Abrocitinib"[Mesh] OR "abrocitinib"[tiab] OR "PF-04965842"[tiab] OR "JAK1 inhibitor"[tiab] OR "Janus kinase 1 inhibitor"[tiab])) AND ("Dermatitis, Atopic"[Mesh] OR "atopic dermatitis"[tiab] OR "eczema"[tiab] OR "moderate-to-severe atopic dermatitis"[tiab] OR "chronic eczema"[tiab])) AND (("Randomized Controlled Trial"[Publication Type] OR "randomized"[tiab] OR "randomised"[tiab] OR "placebo-controlled"[tiab] OR "clinical trial"[tiab]))) Filters: from inception to August 21, 2025. |
| Embase | ('abrocitinib'/exp OR abrocitinib:ti,ab OR 'PF-04965842':ti,ab OR 'jak1 inhibitor':ti,ab OR 'janus kinase 1 inhibitor':ti,ab) AND ('atopic dermatitis'/exp OR 'eczema'/exp OR 'atopic dermatitis':ti,ab OR eczema:ti,ab OR 'moderate-to-severe atopic dermatitis':ti,ab OR 'chronic eczema':ti,ab) AND ('randomized controlled trial'/exp OR random*:ti,ab OR placebo:ti,ab OR 'clinical trial':ti,ab) Limits: database inception to August 21, 2025. |
| Web of Science | TS=(abrocitinib OR "PF-04965842" OR "JAK1 inhibitor" OR "Janus kinase 1 inhibitor") AND TS=("atopic dermatitis" OR eczema OR "moderate-to-severe atopic dermatitis" OR "chronic eczema") AND TS=("randomized controlled trial" OR randomized OR randomised OR placebo OR "clinical trial") Timespan: inception to August 21, 2025. Indexes: SCI-EXPANDED, SSCI, A&HCI, CPCI-S, CPCI-SSH. |
| Cochrane Library | (abrocitinib OR "PF-04965842" OR "JAK1 inhibitor" OR "Janus kinase 1 inhibitor") in Title Abstract Keyword AND ("atopic dermatitis" OR eczema OR "moderate-to-severe atopic dermatitis" OR "chronic eczema") in Title Abstract Keyword AND ("randomized controlled trial" OR randomized OR randomised OR placebo OR "clinical trial") in Title Abstract Keyword. Search limited from inception to August 21, 2025. |
